# An evolutionary algorithm for multi-objective optimization of freshwater consumption in textile dyeing industry

**DOI:** 10.7717/peerj-cs.932

**Published:** 2022-03-22

**Authors:** Ihsan Elahi, Hamid Ali, Muhammad Asif, Kashif Iqbal, Yazeed Ghadi, Eatedal Alabdulkreem

**Affiliations:** 1 Department of Computer Science, National Textile University, Faisalabad, Punjab, Pakistan; 2 Department of Computational Sciences, The University of Faisalabad (TUF), Faisalabad, Punjab, Pakistan; 3 Department of Textile Engineering, National Textile University, Faisalabad, Punjab, Pakistan; 4 Department of Software Engineering/Computer Science, Al Ain University, Al Ain, UAE; 5 Computer Sciences Department, College of Computer and Information Sciences, Princess Nourah bint Abdulrahman University (PNU), Riyadh, Saudi Arabia

**Keywords:** Optimization, Optimization problems, Algorithms, Evolutionary algorithms, Textile dyeing industry

## Abstract

Optimization is challenging even after numerous multi-objective evolutionary algorithms have been developed. Most of the multi-objective evolutionary algorithms failed to find out the best solutions spread and took more fitness evolution value to find the best solution. This article proposes an extended version of a multi-objective group counseling optimizer called MOGCO-II. The proposed algorithm is compared with MOGCO, MOPSO, MOCLPSO, and NSGA-II using the well-known benchmark problem such as Zitzler Deb Thieler (ZDT) function. The experiments show that the proposed algorithm generates a better solution than the other algorithms. The proposed algorithm also takes less fitness evolution value to find the optimal Pareto front. Moreover, the textile dyeing industry needs a large amount of fresh water for the dyeing process. After the dyeing process, the textile dyeing industry discharges a massive amount of polluted water, which leads to serious environmental problems. Hence, we proposed a MOGCO-II based optimization scheduling model to reduce freshwater consumption in the textile dyeing industry. The results show that the optimization scheduling model reduces freshwater consumption in the textile dyeing industry by up to 35% compared to manual scheduling.

## Introduction

Optimization is a well-known technique to find the optimal solution to a complex problem (Eita & Fahmy, 2010; Mirjalili & Lewis, 2016). There are numerous problems in real life that can be categorized as complex problems (Pham et al., 2006). It is tough to solve these problems using traditional algorithms. Optimization plays an essential role in solving these complex problems due to resource efficiency. These problems have many scenarios where an objective can be converted into an optimization problem. Optimization problems are classified into single-objective and multi-objective problems (Ali & Khan, 2012, 2013a). Single objective optimization problems are those problems that have only one objective to minimize/maximize. Multi-objective optimization problems can be categorized into two or more goals to minimize/maximize. Evolutionary algorithms can quickly solve optimization problems (Anchor et al., 2002; Mugambi & Hunter, 2003).

Evolutionary algorithms are generic population-based and evolution-based metaheuristic algorithms (Hruschka, Campello & Freitas, 2009; Slowik & Kwasnicka, 2020). Evolutionary algorithms are helpful to minimize/maximize real-world problems (complex problems). These algorithms have gained popularity in the last decade due to several advantages in the optimization field compared to traditional techniques (Slowik & Kwasnicka, 2020; Rajabi & Witt, 2020).

For multi-objective optimization problems (Gómez et al., 2013; Rizk-Allah, Hassanien & Slowik, 2020), the researchers proposed many multi-objective evolutionary algorithms such as Multi-Objective Particle Swarm Optimizer (MOPSO) (Dou et al., 2021), Non-Dominated Sorting Genetic Algorithm II (NSGA-II) (Cai et al., 2019; Yi et al., 2020), Multi-Objective Comprehensive Learning Particle Swarm Optimizer (MOCLPSO) (Zhang, Wang & Ye, 2018), Attributed Multi-Objective Comprehensive Learning Particle Swarm Optimizer (AMOCLPSO) (Ali & Khan, 2013b), and the Multi-Objective Group Counseling Optimizer (MOGCO) (Ali & Khan, 2013a). However, these algorithms have failed to find out the best spread of solutions and convergence near the actual Pareto optimal front. And take more fitness evolution value to find out the best solution.

Moreover, the textile dyeing industry is an essential unit because it converts the raw material of textile fibers into finished products. The dye used in this process depends on the type of material and the specific requirement of the final product. In the dyeing industry, the dyeing process needs more chemicals that’s why the wastewater from the dyeing process is more hazardous than the other processes.

**Figure 1:**
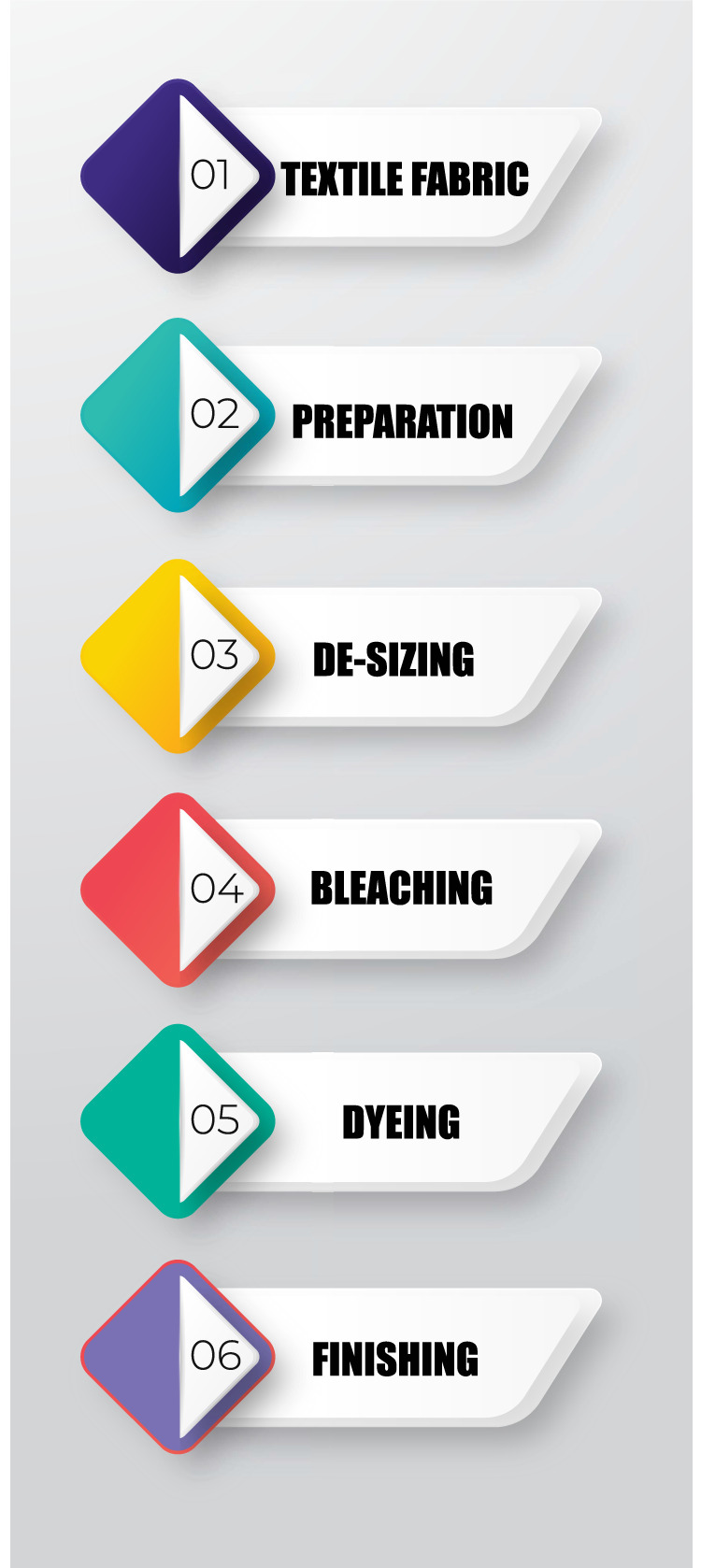
Textile dyeing process.

The dyeing process is the most common and essential factor of textile products in successful trading. Instead, with time, the dyeing industry still uses the oldest methods and techniques to dye the fabrics. The modern dyeing process consists of different steps based on fiber nature, dye properties, and pigments used for the materials like (Zhou et al., 2017; Hsu et al., 2009): structure of the chemical, commercial availability, classification, fixing features, economic considerations, etc. In the dyeing process, fresh water is used to clean, dye, and apply the auxiliary chemicals to the fabrics and rinsing. The dyeing process depends on three steps, as shown in Fig. 1.

The dyeing industry consumes a large quantity of freshwater and produces much-polluted water from the different preparations, including dyeing and finishing steps. In addition, the contaminated water has riches from the dyeing process with colors and chemicals (Jiang et al., 2010).

This article proposes an extended version of the MOGCO algorithm called Multi-Objective Group Counseling Optimizer II (MOGCO-II) based optimization model. The main contributions of this study are as follows:

- We proposed the new version of the MOGCO algorithm called MOGCO-II to optimize fresh water consumption in the textile dyeing industry.
- We tested the proposed algorithm using several standard benchmark functions: ZDT1, ZDT2, ZDT3, ZDT4, ZDT6, and performance metrics: generation distance metric and diversity distance metric (Xu et al., 2020).
- The comparisons of MOGCO-II were performed with several evolutionary multi-objective (EMO) algorithms: MOPSO (Dou et al., 2021), MOCLPSO (Zhang, Wang & Ye, 2018), NSGA-II (Yi et al., 2020), and MOGCO (Ali & Khan, 2013a).

The rest of the paper is organized as follows: “Related Work” explains the related work. “Research Methodology” describes the methodology used for this research work. “Proposed Algorithm MOGCO-II” describes the proposed algorithm. “Experiments and Results” presents the experiments and results. “Optimization of Freshwater Consumption in Textile Dyeing Industry” describes the Optimization model for the dyeing industry, and “Conclusion” concludes this research work.

## Related Work

Yi et al. (2020) proposed a new heuristic-based multi-objective optimization algorithm called non-dominated sorting genetic algorithm II (NSGA-II). NSGA-II is used for non-dominated problems, but it can not solve the issues dominated. It also uses more time and fitness evolution values to determine the optimal results.

Dou et al. (2021) proposed a new version of particle swarm optimizer (PSO) called multi-objective particle swarm optimizer (MOPSO). However, the PSO (Coello, Pulido & Lechuga, 2004; Kennedy & Eberhart, 1995) only solves the non-dominated complex multi-objective optimization problems. So, MOPSO integrated the concept of Pareto dominance that’s why MOPSO successfully solved the dominated complex problems. However, the MOPSO is a time-consuming algorithm, and it takes more fitness evolution value to find out the optimal results for the multi-objective optimization problems.

Zhang, Wang & Ye (2018) proposed the new version of comprehensive particle swarm optimizer (CLPSO) called multi-objective complete learning particle swarm optimizer (MOCLPSO) (Huang, Suganthan & Liang, 2006; El-Zonkoly, Saad & Khalil, 2013). The CLPSO is used for non-dominated problems (Liang, Zhigang & Zhihui, 2010). The MOCLPSO solves both dominated and non-dominated problems. The results show that MOCLPSO finds a much better spread of solutions near the actual Pareto front and faster convergence to the true Pareto front than other algorithms. But MOCLPSO is also a time-consuming algorithm and takes more fitness evolution value to find the optimal solution of complex problems.

The researchers introduced a new multi-objective algorithm of PSO called AMOCLPSO (Ali & Khan, 2013b). A particle used its personal best and local or global best positions by linear summation in particle swarm optimization (PSO). However, finding the best local or global locations is time-consuming in complex problems. AMOCLPSO overcome this problem. This technique does not use local or global best positions to modify a particle’s velocity; instead, it uses the best view of a randomly selected particle from the whole population to update each dimension’s rate.

The researchers introduced a technique called Multi-Object Group Counseling Optimizer (MOGCO) in Ali & Khan (2013a) in which they extended the existing Group Counseling Optimizer (GCO) (Eita, Shoukry & Iba, 2014). GCO is only used for single-object optimization problems, but MOGCO is used for multi-object optimization problems. MOGCO provides a dominant solution for multi-object optimization problems. MOGCO is the only algorithm that can sleeve the entire Pareto front for all the test functions used for testing the algorithms.

Zhou et al. (2017) proposed an optimization model based on a genetic algorithm to optimize fresh water consumption in the textile dyeing industry. It aimed to reduce freshwater consumption in the textile dyeing industry by optimizing the production schedule based on the dyeing color and depth. The optimization model is developed by using Matlab and establishing a database of different orders of different orders of textile companies of China. The proposed model is compared to traditional production scheduling and the results show that the proposed optimization model could reduce freshwater consumption by about 18% to 21%.

Jiang et al. (2010) proposed an optimization production scheduling based on genetic algorithms for this research using a Chinese enterprise’s data set of dyeing department. This research minimized freshwater consumption by 20% to 21% and reduced production time by 10% to 12%. So, through this research, control of the consumption of fresh water reduces the quantity of polluted water. This leads to the arrest of the water problems of the environment.

Hsu et al. (2009) proposed a genetic algorithm-based production scheduling technique for optimizing the yarn industry’s production scheduling. This research used the data set of the yarn dyeing department of a chines based enterprise. This reduces freshwater consumption in the dyeing process of yarn, eliminating the quantity of polluted water and water environmental problems.

The comparative analysis of selected literature is shown in Table 1.

**Table 1:** Comparative analysis of selected literature.

SR #	Study reference	Domain	Techniques	Research gap
1	Ali & Khan (2013a)	Optimization	Single-Objective and Multiobjective Optimization, Pareto Optimal Set, Pareto Front, Performance Matrices, Multiobjective Group Counseling Optimizer, Random Selection.	It is failed to find out best spread of solution and convergence near the true pareto optimal front.
2	Yi et al. (2020)	Optimization	Multi-Objective Optimization, Non-Dominated Problems, Genetic Algorithm.	It takes more fitness evolution value to find out optimal front. Also failed to find out best spread of solution and convergence near the true pareto optimal front.
3	Dou et al. (2021)	Optimization	Multi-Objective Optimization, Dominated Complex Problems, Multi-Objective Particle Swarm Optimizer.	It is a time-consuming algorithm and it takes more fitness evolution value to find out the optimal results for the multi-objective optimization problems.
4	Zhang, Wang & Ye (2018)	Optimization	Multi-Objective Optimization, Dominated Complex Problems, Multi-Objective Comprehensive Particle Swarm Optimizer.	It is also a time-consuming algorithm and takes more fitness evolution value to find the optimal solution of complex problems.
5	Ali & Khan (2013b)	Optimization	Multi-Objective Optimization, Dominated Complex Problems, Attributive Multi-Objective Comprehensive Particle Swarm Optimizer.	This technique does not use local or global best positions to modify a particle’s velocity; instead, it uses the best view of a randomly selected particle from the whole population to update each dimension’s rate.
6	Zhou et al. (2017)	Optimization and Textile Dyeing Industry	Mat Lab, Genetic Algorithm, Production Scheduling Methods,	Data Set of Textile Dyeing Industry China, In this optimization model used the genetic algorithm.
7	Jiang et al. (2010)	Optimization and Dyeing Industry	Mat Lab, Genetic Algorithm, Production Scheduling Methods	Data Set of Textile Dyeing Industry China, In this optimization model used the genetic algorithm.
8	Hsu et al. (2009)	Optimization and Yarn Dyeing Industry	Mat Lab, Genetic Algorithm, Production Scheduling	Data Set of Yarn Dyeing Industry China. In this optimization model used the genetic algorithm

## Research Methodology

The Zitzler Deb Thiele’s (ZDT) family of test functions are used for the experimental results, a popular set of functions for benchmarking the performance of different multi-objective optimization methods. These test functions have some particular types of features that are representative of various real-world optimization problems.

### Test functions and performance metrics

The Zitzler Deb Thiele’s (ZDT) test suite created by Zitzler, Deb & Thiele (2000) is perhaps the most widely used benchmark problem for multiobjective optimization algorithms. ZDT3 is disconnected on both the Pareto optimal set and front, the latter of which consists of one composite convex/concave component and several convex components. It should also be noted that ZDT4 has one parameter of the different domains (Ali & Khan, 2013a), whereas all other parameters have the environment (Dou et al., 2021). Nevertheless, the ZDT problems share many of the characteristics, such as how multimodality can cause Pareto many-to-one problems (ZDT6), multifrontal problems (ZDT4), and so-called disconnected problems (ZDT3). Importantly, all ZDT problems employ only one position parameter, meaning it is a function of only one parameter. The ZDT test suite offers two main advantages: (1) well-defined Pareto optimal fronts, (2) test results are commonly available from various other research papers, which facilitates comparisons with new algorithms. The details of the ZDT test suit are given in Table 2. Two performance metrics are also used to evaluate the algorithm’s results: Generation Distance Metric and Diversity Distance Metric (Navarro-Muñoz et al., 2020).

**Table 2:** Description of ZDT test suit.

Problem	*N*	Variable bounds	Objective functions	Optimal solution	Comments
ZDT1	50	[0,1]	${f_1}\left( x \right) = {x_1}$ ${f_2}\left( x \right) = g\left( x \right)\left[ {1 - \sqrt {\displaystyle{{{x_1}} \over {g\left( x \right)}}} } \right]$ $g\left( x \right) = 1 + 9\left( {\mathop \sum \nolimits_{i = 2}^n {x_i})/(n - 1} \right)$	X_1_ ∈ [0,1] X_i_ = 0 i = 2,…, *n*	Convex
ZDT2	50	[0,1]	${f_1}\left( x \right) = {x_1}$ ${f_2}\left( x \right) = g\left( x \right)\left[1 - \left( \displaystyle{{{x_1}} \over {g\left( x \right)}}\right)^2 \right]$ $g\left( x \right) = 1 + 9\left( {\mathop \sum \nolimits_{i = 2}^n {x_i})/(n - 1} \right)$	X_1_ ∈ [0,1] X_i_ = 0 i = 2,…, *n*	Nonconvex
ZDT3	50	[0,1]	${f_1}\left( x \right) = {x_1}$ ${f_2}\left( x \right) = g\left( x \right)\left[ {1 - \sqrt {\displaystyle{{{x_1}} \over {g\left( x \right)}}} - \displaystyle{{{x_1}} \over {g\left( x \right)}}\sin (10\pi {x_1}} )\right]$ $g\left( x \right) = 1 + 9\left( {\mathop \sum \nolimits_{i = 2}^n {x_i})/(n - 1} \right)$	X_1_ ∈ [0,1] X_i_ = 0 i = 2,…, *n*	Convex, Disconnected
ZDT4	50	X_1_ ∈ [0,1] X_i_ ∈ [–5,5] i = 2,…, *n*	${f_1}\left( x \right) = {x_1}$ ${f_2}\left( x \right) = g\left( x \right)\left[ {1 - \sqrt {\displaystyle{{{x_1}} \over {g\left( x \right)}}} } \right]$ $g\left( x \right) = 1 + 10\left( {n - 1} \right) + \mathop \sum \nolimits_{i = 2}^n [x_i^2 - 10\cos (4\pi {x_i})]$	X_1_ ∈ [0,1] X_i_ = 0 i = 2,…, *n*	Convex
ZDT6	50	[0,1]	${f_1}\left( x \right) = 1 - \exp ( - 4{x_i}){\sin ^6}(6\pi {x_1})$ ${f_2}\left( x \right) = g\left( x \right)\left[1 - \left( \displaystyle{{{f_1}\left( x \right)} \over {g\left( x \right)}}\right)^2 \right]$ $g\left( x \right) = 1 + 9{[(\mathop \sum \nolimits_{i = 2}^n {x_i})/\left( {n - 1} \right)]^{0.25}}$	X_1_ ∈ [0,1] X_i_ = 0 i = 2,…, *n*	Non Convex, Nonuniformly Space

### Establishment of the database for production scheduling

We established a database with the following data: order, dye, auxiliary, freshwater, wastewater data. In addition, energy consumption data: based on the investigation of any dyeing enterprise. We obtained the order data from the ERP department, dye data, and auxiliary data from the department of production measurements. Data for energy consumption and water consumption data have been elicited from the department of measuring equipment (dyeing vessels) (Zhou et al., 2017).

Order data contain fabric type, color, depth of color, the material’s weight, delivery date, and other information. Dye data have dye rate and COD of dye. Auxiliary data provide extra details of chemical composition. Energy data may consume vessels’ energy, detail dyeing machine, volume, bath ratio, and temperature. Water consumption data contain the detail of discharge wastewater at every step, COD, size, color, electrical conductivity, temperature, and the suspended solids. The above mentioned data and parameters can be changed based on enterprise investigation.

### Assumptions used for production scheduling

Some assumptions help optimize production scheduling in the textile dyeing industry (Zhou et al., 2017).

- Simplify the problem for the orders and vessels by creating one-to-one relations among ships and rules.
- Obtain the following data: the data of order, the data of dye, the data of auxiliary, the data of freshwater, the data of wastewater, and the data of energy consumption from the database.
- It does not need washing when switching the order between the same and dark colors.
- The fabric, which has the same color but different depth, needs a 5 m^3^ volume of freshwater.
- The fabric, which has a different color and depth, needs a 10 m^3^ volume of freshwater.

### Scheduling methods in dyeing enterprises

There are the following scheduling methods in dyeing enterprises: (Jiang et al., 2010; Hsu et al., 2009).

- Important parameters: The delivery time is the most critical parameter for the order. For urgent orders and special orders (orders for unique customers), the enterprises can sacrifice profit to achieve customer relations and reputation.
- Orders consolidation: The same cloth color, depth of color, cloth type, and production process should be put together in the same dyeing vessel.
- Sorting orders: Firstly, orders should be sorted based on different colors. After grouping based on colors, sort each group of orders based on color depth from light to dark.

### Optimizing production scheduling strategies

There are following Optimizing Production Scheduling Strategies:

- The dyeing vessels can be used for the production of any order. If the order’s size is greater than the capacity of vessel production, then the order should be divided into multiple parts. If the orders are small and have the same color, type of cloth, and depth of color, we should put these orders simultaneously in the same vessel (Zhou et al., 2017).
- Parameters of the order are: order number, customer name, delivery date, the color of cloth, depth of color and weight of the material, Parameters of the quantity of freshwater and wastewater, electrical conductivity, COD, volume, temperature and suspended slides, these all parameters are obtained from the DBMS.
- The fabric colors are divided into eight different colors: black, blue, cyan, green, orange, purple, red, and yellow. Every color further can be divided into light, medium, and dark colors based on color depth (Zhou et al., 2017).
- An order almost covers 10 to 12 h in actual production. But we will assume a fixed digit for every order, which means that the order will be switched after a fixed duration of time. So the order changing completely depends on the time interval used for each order (Zhou et al., 2017).
- The rinsing water can reuse directly, and the light-polluted wastewater and heavily polluted wastewater must be treated separately (Zhou et al., 2017).

## Proposed Algorithm MOGCO-II

**Figure 2:**
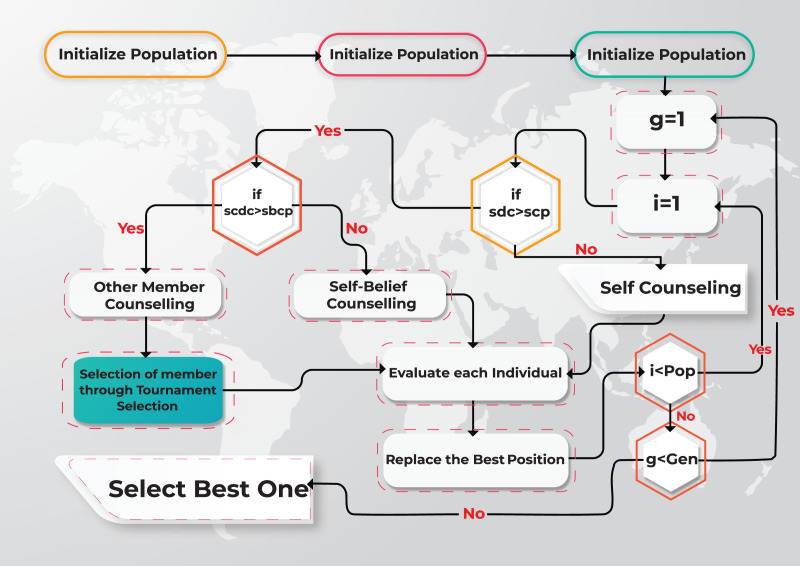
Flow chart of the proposed MOGCO-II algorithm.

MOGCO successfully solved the multi-objective optimization problems (Ali & Khan, 2013a). But it has failed to find out the best spread of solutions and convergence near the actual Pareto optimal front, taking more fitness evolution value to find out the optimal show. To solve these problems, a Pareto dominance-based Multi-Objective Group Counseling Optimizer II (MOGCO-II) algorithm is presented to handle multi-objective optimization problems. The flow chart of the proposed MOGCO-II algorithm is shown in Fig. 2.

MOGCO-II is the latest version of MOGCO. However, MOGCO-II only updated the part of other members consoling. In this portion, the MOGCO used a random selection technique (Zhou, Liu & Chen, 2011), but MOGCO-II has used the tournament selection technique instead of the random selection technique. After this change, MOGCO-II produces the best solution spread and convergence near the actual Pareto optimal front.

**Figure 3:**
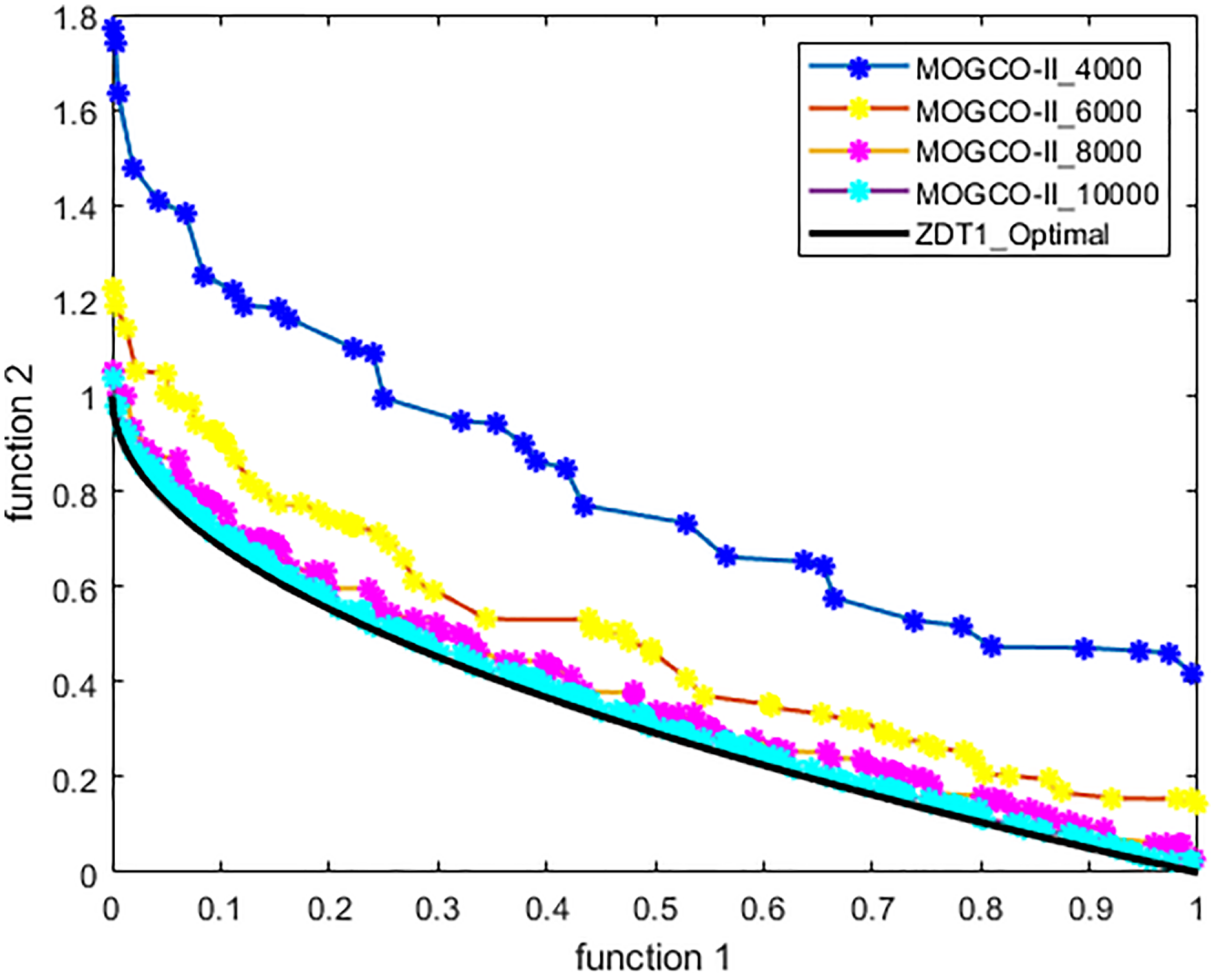
Pareto front of MOGCO-II for ZDT1 on fitness evolution values 4,000, 6,000, 8,000 and 10,000.

**Figure 4:**
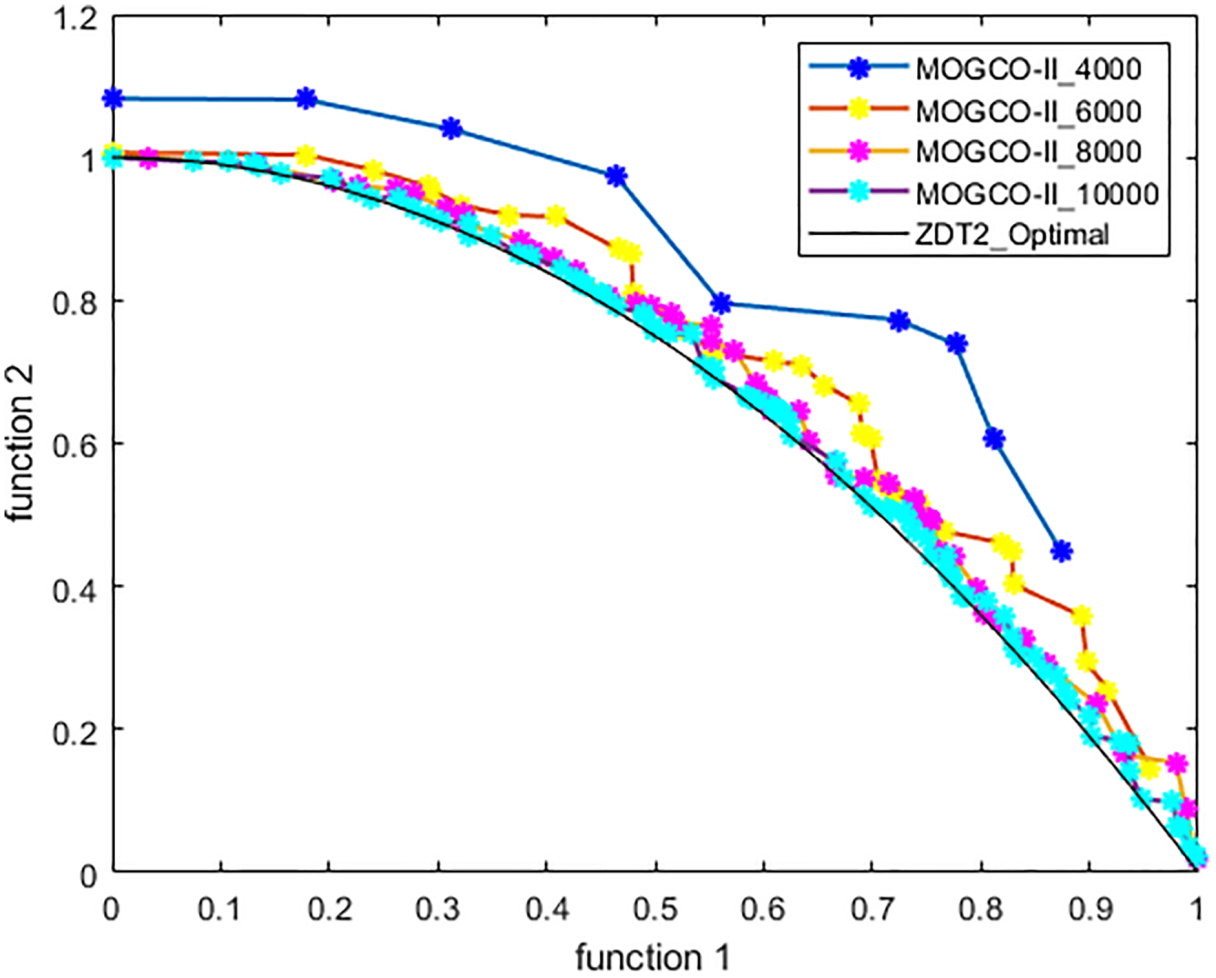
Pareto front of MOGCO-II for ZDT2 on fitness evolution values 4,000, 6,000, 8,000 and 10,000.

**Figure 5:**
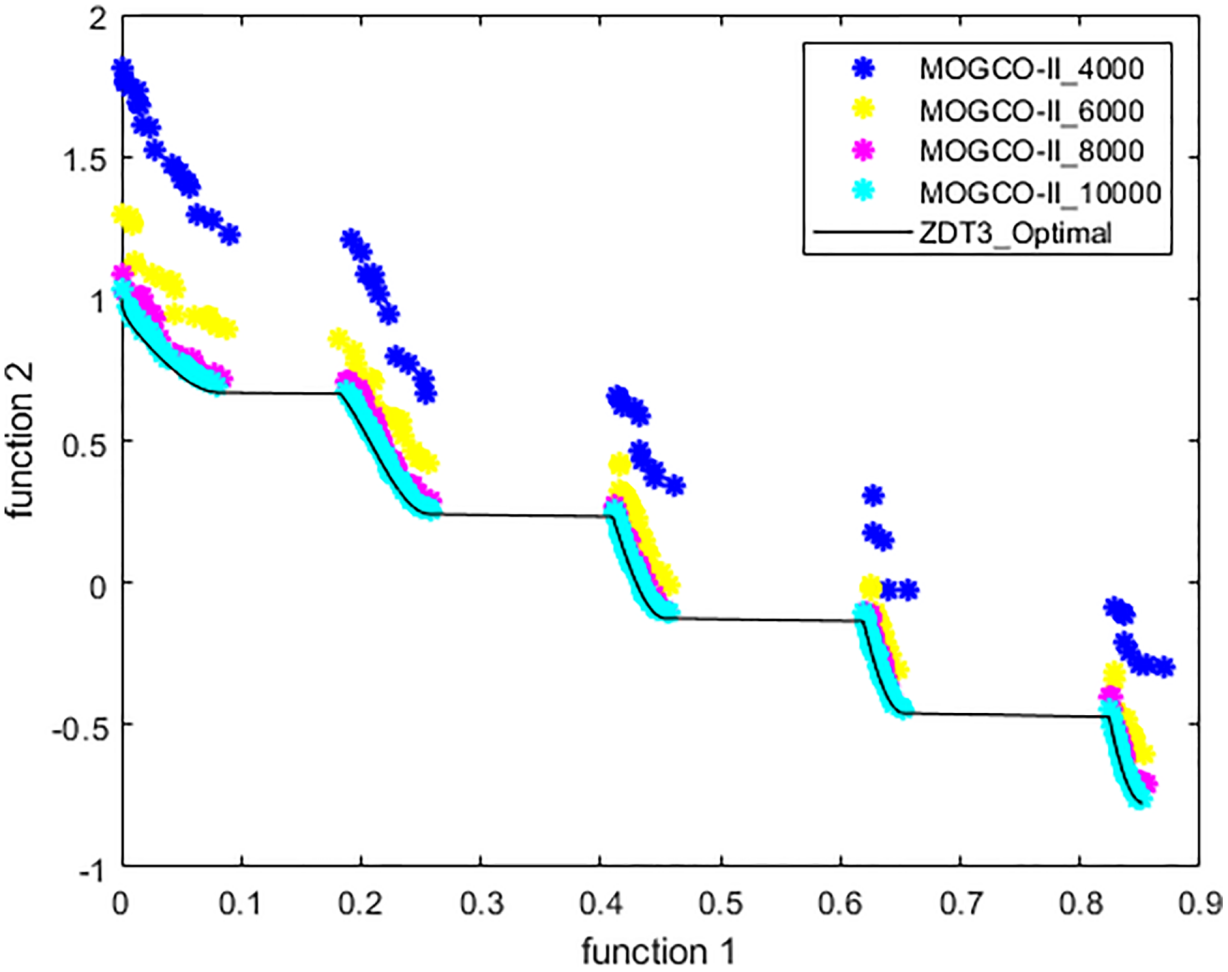
Pareto front of MOGCO-II for ZDT3 on fitness evolution values 4,000, 6,000, 8,000 and 10,000.

**Figure 6:**
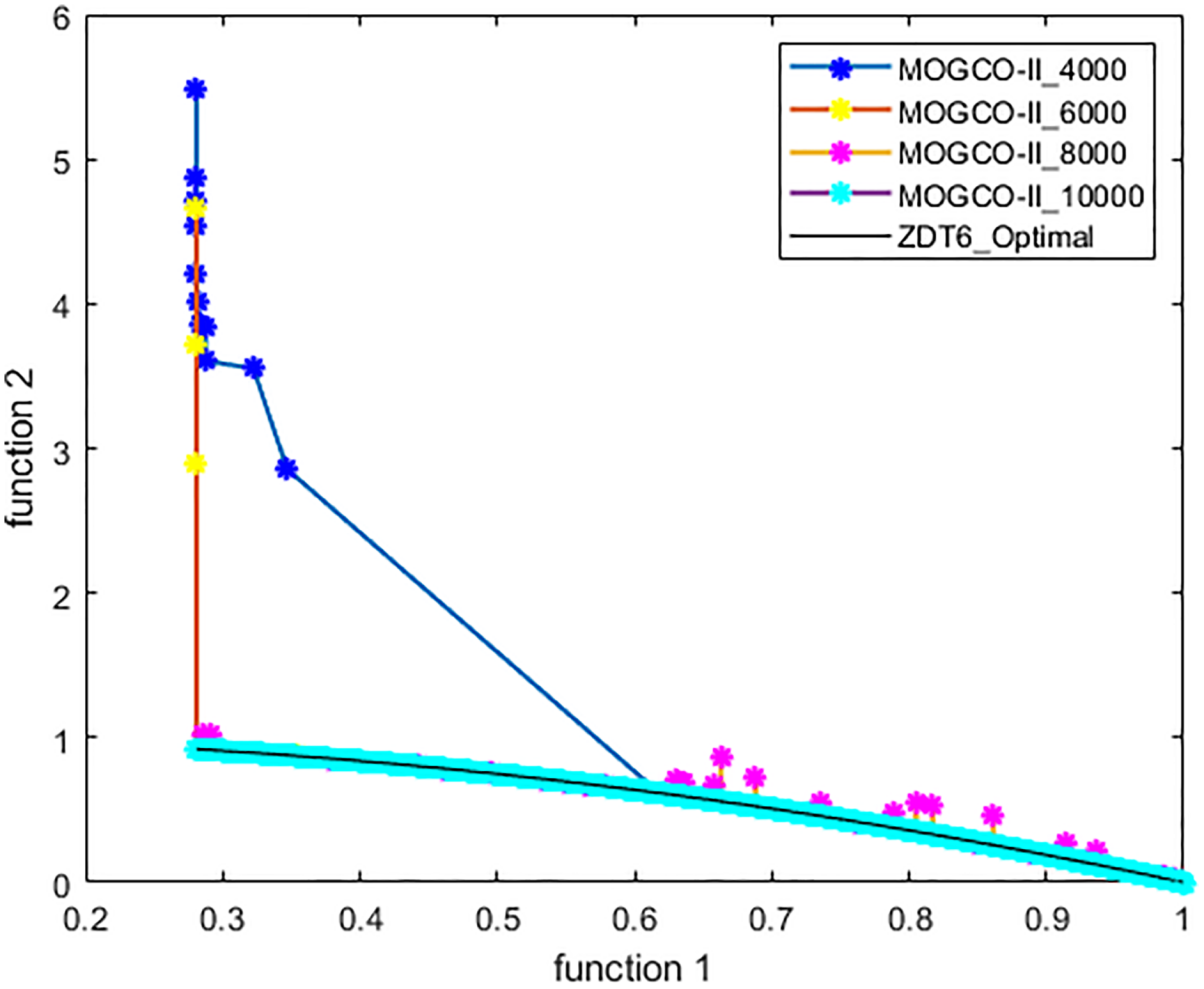
Pareto front of MOGCO-II for ZDT4 on fitness evolution values 15,000, 20,000, 25,000 and 30,000.

**Figure 7:**
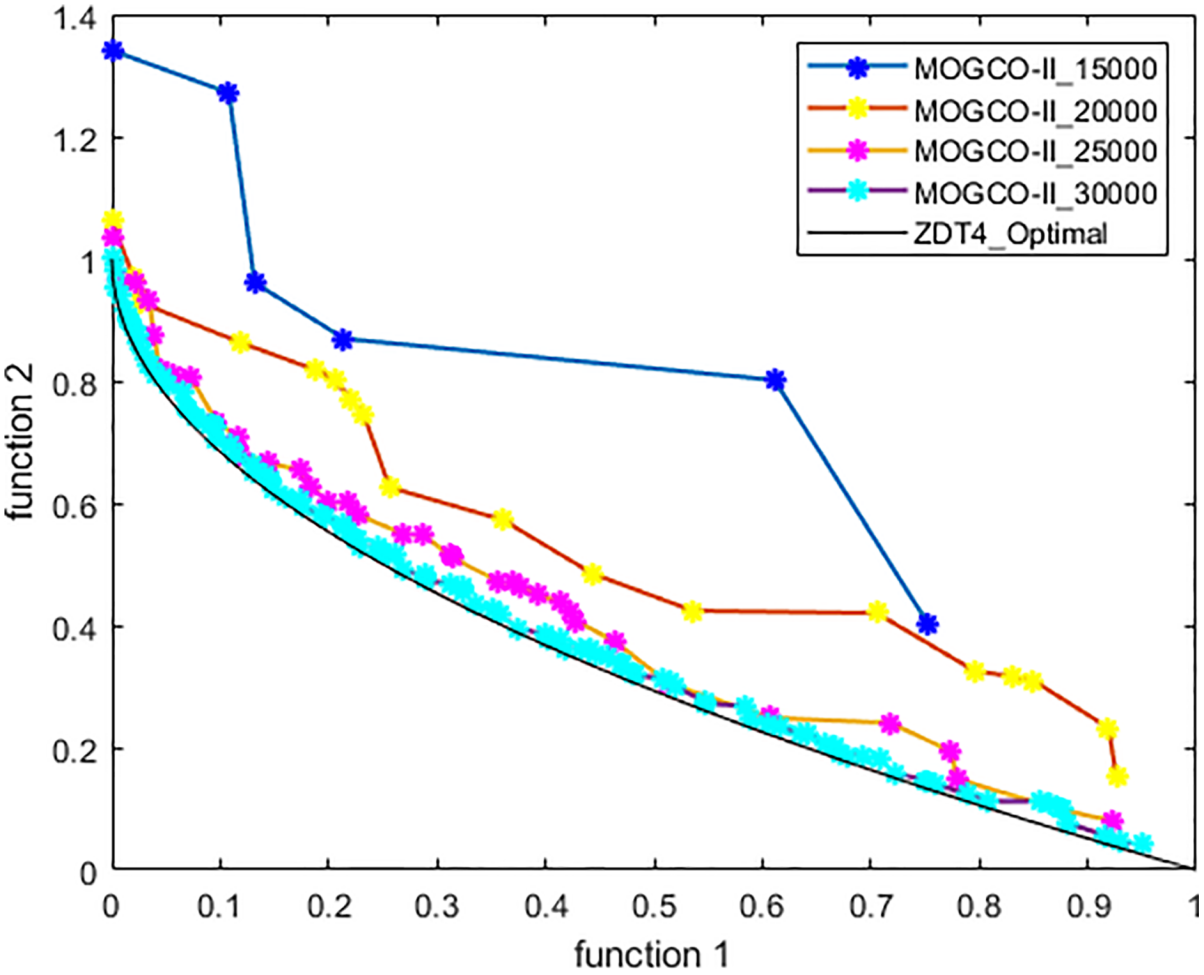
Pareto front of MOGCO-II for ZDT6 on fitness evolution values 4,000, 6,000, 8,000 and 10,000.

Figures 3–6 show the Pareto fronts of ZDT1, ZDT2, ZDT3, and ZDT6, respectively, for MOGCO-II on fitness evolution values 4,000, 6,000, 8,000, and 10,000. Figure 7 shows the Pareto fronts of ZDT4 for MOGCO-II on fitness evolution values 15,000, 20,000, 25,000, and 30,000.

The results show that as we increase the fitness evolution value, the optimal value of the algorithm is not trapped. The increment in the fitness revolution values does not affect the optimality of the algorithm. The algorithm consistently achieved higher optimal values. The results also show that for the test function ZDT1, ZDT2, ZDT3, and ZDT6, the proposed algorithm generates the best Pareto front, the best spread of solutions, and convergence near the true Pareto optimal front on fitness evolution value 10,000, and for ZDT4 generates the best Pareto front, the best stretch of solutions, an intersection near the true Pareto optimal front on fitness evolution value 30,000.

## Experiments and Results

The comparison of the proposed algorithm is made with five well-known evolutionary algorithms: MOPSO, MOCLPSO, AMOCLPSO, NSGA-II, and MOGCO. In “Research Methodology”, the results show that the MOGCO-II provides the best Pareto front, the best spread of solutions, and convergence near the true Pareto optimal front on fitness evolution value 10,000 for each ZDT1, ZDT2, ZDT3, ZDT6, and 30,000 for ZDT4. Hence, the comparisons of MOGCO-II performed on 10,000 fitness evolution for ZDT1, ZDT2, ZDT3, ZDT6, and 30,000 for ZDT4 with MOPSO, MOCLPSO, NSGA-II, and MOGCO.

### Test function 1 (ZDT1)

The ZDT1 benchmark function is used for the first experiment. Tables 3 and 4 show the performance metric’s comparison of MOGCO, MOCLPSO, MOPSO, NSGA-II, and MOGCO-II. The results show that MOGCO-II performs better than the existing algorithms.

**Table 3:** Results of generation distance metric for ZDT1.

FE	Algorithms	Best	Worst	Average	Median	Standard deviation
10,000	MOPSO NSGA-II MOGCO MOCLPSO MOGCO-II	4.85 × 10^–01^ 3.48 × 10^–02^ 5.71 × 10^–02^ 4.82 × 10^–01^ 2.73 × 10^–02^	1.32 × 10^+00^ 1.27 × 10^+00^ 1.01 × 10^–01^ 2.07 × 10^+00^ 4.74 × 10^–02^	8.57 × 10^–01^ 1.42 × 10^–01^ 7.98 × 10^–02^ 1.30 × 10^+00^ 3.55 × 10^–02^	8.40 × 10^–01^ 8.84 × 10^–02^ 8.07 × 10^–02^ 1.25 × 10^+00^ 3.57 × 10^–02^	1.97 × 10^–01^ 1.98 × 10^–01^ 1.06 × 10^–02^ 3.81 × 10^–01^ 3.60 × 10^–03^

**Table 4:** Results of diversity metric for ZDT1.

FE	Algorithms	Best	Worst	Average	Median	Standard deviation
10,000	MOPSO NSGA-II MOGCO MOCLPSO MOGCO-II	6.39 × 10^–01^ 3.09 × 10^–01^ 1.20 × 10^–01^ 7.99 × 10^–01^ 4.46 × 10^–02^	9.03 × 10^–01^ 7.96 × 10^–01^ 2.81 × 10^–01^ 9.85 × 10^–01^ 2.13 × 10^–01^	8.12 × 10^–01^ 4.54 × 10^–01^ 1.92 × 10^–01^ 9.14 × 10^–01^ 1.09 × 10^–01^	8.16 × 10^–01^ 4.29 × 10^–01^ 1.85 × 10^–01^ 9.19 × 10^–01^ 1.04 × 10^–01^	4.68 × 10^–02^ 9.23 × 10^–02^ 4.08 × 10^–02^ 3.84 × 10^–02^ 3.43 × 10^–02^

**Figure 8:**
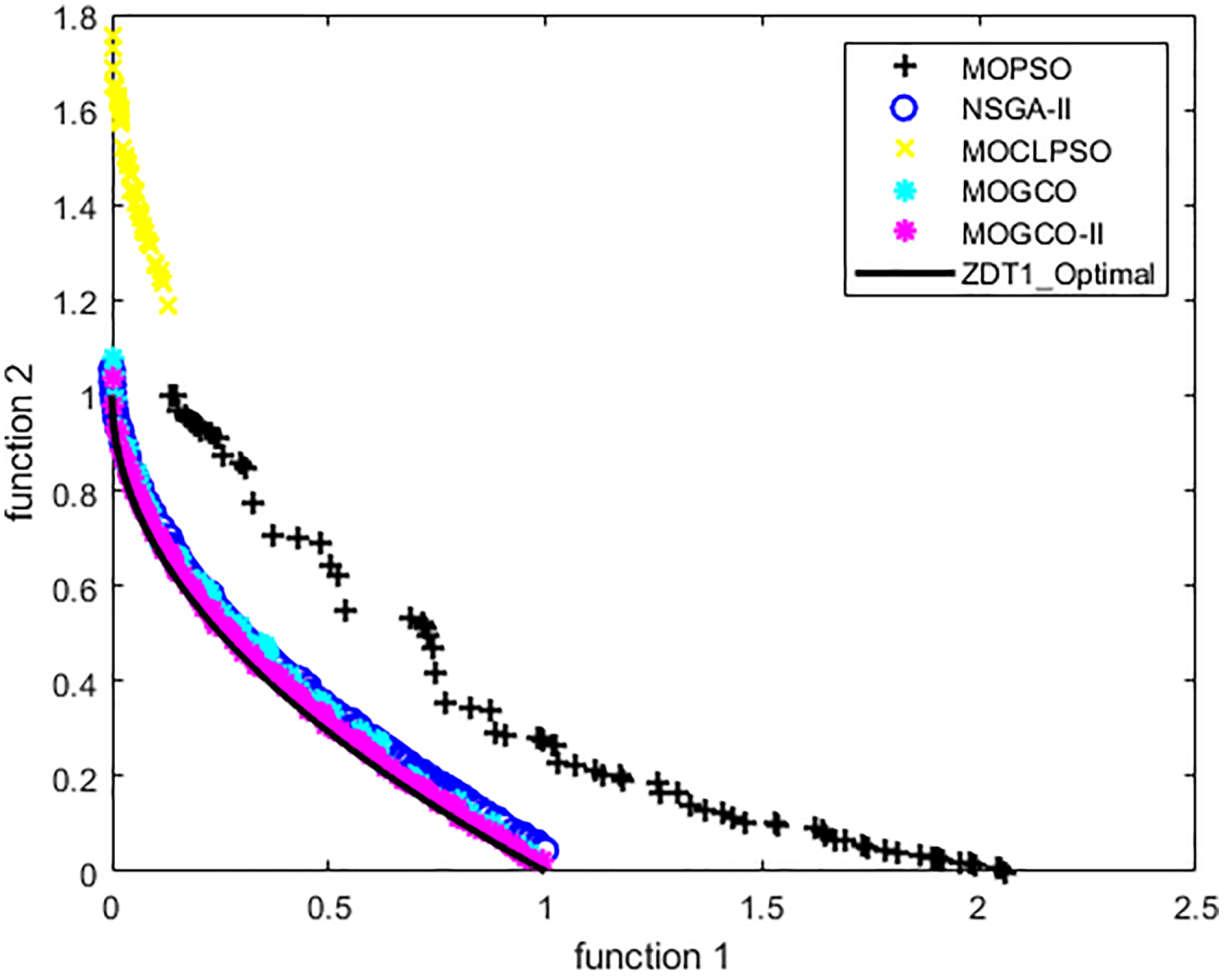
Pareto front of MOPSO, NSGA-II, MOGCO, MOCLPSO, and MOGCO-II for ZDT1.

Figure 8 presents the results produced by MOCLPSO, MOGCO, MOPSO, NSGA-II, and MOGCO-II. The results show the best Pareto fronts have by the algorithms after 50 runs at 10,000 fitness evaluations for test function ZDT1. In addition, the results show that MOGCO-II provides the best Pareto front, the best spread of solutions, and convergence near the actual Pareto optimal front compared to other algorithms.

### Test function 2 (ZDT2)

The ZDT2 benchmark function is used for the second experiment. Tables 5 and 6 show the performance metric’s comparison of MOCLPSO, MOPSO, MOGCO, NSGA-II, and the proposed algorithm. The tables show that MOGCO-II performs better than the existing algorithms.

**Table 5:** Results of generation distance metric for ZDT2.

FE	Algorithms	Best	Worst	Average	Median	Standard deviation
10,000	MOPSO NSGA-II MOGCO MOCLPSO MOGCO-II	0.00 × 10^+00^ 4.62 × 10^–02^ 6.55 × 10^–02^ 0.00 × 10^+00^ 1.89 × 10^–02^	6.15 × 10^–01^ 1.49 × 10^+00^ 1.59 × 10^–01^ 2.99 × 10^–01^ 6.96 × 10^–02^	4.76 × 10^–01^ 2.67 × 10^–01^ 1.14 × 10^–01^ 1.01 × 10^–01^ 4.18 × 10^–02^	0.00 × 10^+00^ 1.73 × 10^–01^ 1.17 × 10^–01^ 0.00 × 10^+00^ 4.26 × 10^–02^	1.27 × 10^–01^ 3.11 × 10^–01^ 2.08 × 10^–02^ 4.35 × 10^–02^ 1.15 × 10^–02^

**Table 6:** Results of diversity metric for ZDT2.

FE	Algorithms	Best	Worst	Average	Median	Standard deviation
10,000	MOPSO NSGA-II MOGCO MOCLPSO MOGCO-II	7.33 × 10^–01^ 4.62 × 10^–01^ 1.91 × 10^–01^ 9.69 × 10^–01^ 3.72 × 10^–02^	1.10 × 10^+00^ 1.00 × 10^+00^ 5.39 × 10^–01^ 1.01 × 10^+00^ 2.72 × 10^–01^	9.77 × 10^–01^ 8.01 × 10^–01^ 3.18 × 10^–01^ 9.98 × 10^–01^ 1.64 × 10^–01^	1.00 × 10^+00^ 9.40 × 10^–01^ 2.99 × 10^–01^ 1.00 × 10^+00^ 1.55 × 10^–01^	6.56 × 10^–02^ 2.01 × 10^–01^ 6.87 × 10^–02^ 6.37 × 10^–03^ 5.77 × 10^–02^

**Figure 9:**
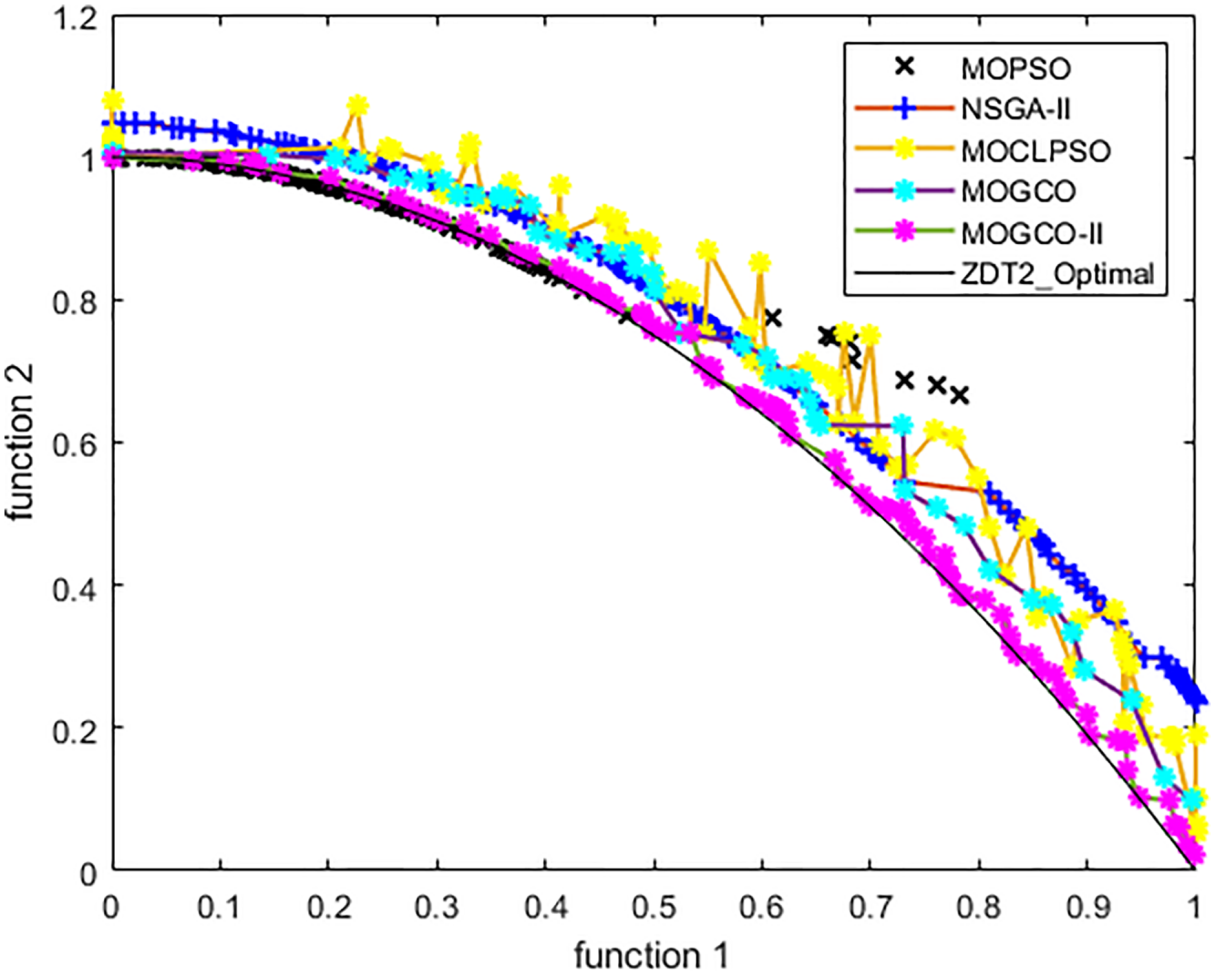
Pareto front of MOPSO, NSGA-II, MOGCO, MOCLPSO, and MOGCO-II for ZDT2.

Figure 9 presents the results produced by MOCLPSO, MOGCO, MOPSO, NSGA-II, and the proposed algorithm. The results show the best Pareto fronts have by the algorithms after 50 runs at 10,000 fitness evaluations for the test function ZDT2. In addition, the results show that MOGCO-II provides the best Pareto front, the best spread of solutions, and convergence near the actual Pareto optimal front compared to other algorithms.

### Test function 3 (ZDT3)

The ZDT3 benchmark function is used for the third experiment. Tables 7 and 8 show the performance metric’s comparison of MOCLPSO, MOPSO, MOGCO, NSGA-II, and the proposed algorithm. The tables show that MOGCO-II performs better than the existing algorithms.

**Table 7:** Results of generation distance metric for ZDT3.

FE	Algorithms	Best	Worst	Average	Median	Standard deviation
10,000	MOPSO NSGA-II MOGCO MOCLPSO MOGCO-II	3.94 × 10^–01^ 3.52 × 10^–02^ 2.84 × 10^–02^ 7.09 × 10^–01^ 1.14 × 10^–02^	1.88 × 10^+00^ 1.27 × 10^+00^ 8.67 × 10^–02^ 2.35 × 10^+00^ 3.89 × 10^–02^	1.14 × 10^+00^ 1.89 × 10^–01^ 5.02 × 10^–02^ 1.75 × 10^+00^ 2.23 × 10^–02^	1.13 × 10^+00^ 1.23 × 10^–01^ 4.95 × 10^–02^ 1.83 × 10^+00^ 2.10 × 10^–02^	3.27 × 10^–01^ 2.40 × 10^–01^ 1.31 × 10^–02^ 4.30 × 10^–01^ 6.69 × 10^–03^

**Table 8:** Results of diversity metric for ZDT3.

FE	Algorithms	Best	Worst	Average	Median	Standard deviation
10,000	MOPSO NSGA-II MOGCO MOCLPSO MOGCO-II	7.85 × 10^–01^ 5.43 × 10^–01^ 8.99 × 10^–02^ 7.81 × 10^–01^ 5.53 × 10^–02^	9.93 × 10^–01^ 8.39 × 10^–01^ 2.03 × 10^–01^ 1.02 × 10^+00^ 1.54 × 10^–01^	8.98 × 10^–01^ 6.59 × 10^–01^ 1.46 × 10^–01^ 8.87 × 10^–01^ 1.01 × 10^–01^	8.94 × 10^–01^ 6.50 × 10^–01^ 1.46 × 10^–01^ 8.92 × 10^–01^ 1.01 × 10^–01^	4.99 × 10^–02^ 7.12 × 10^–02^ 2.82 × 10^–02^ 5.75 × 10^–02^ 1.99 × 10^–02^

**Figure 10:**
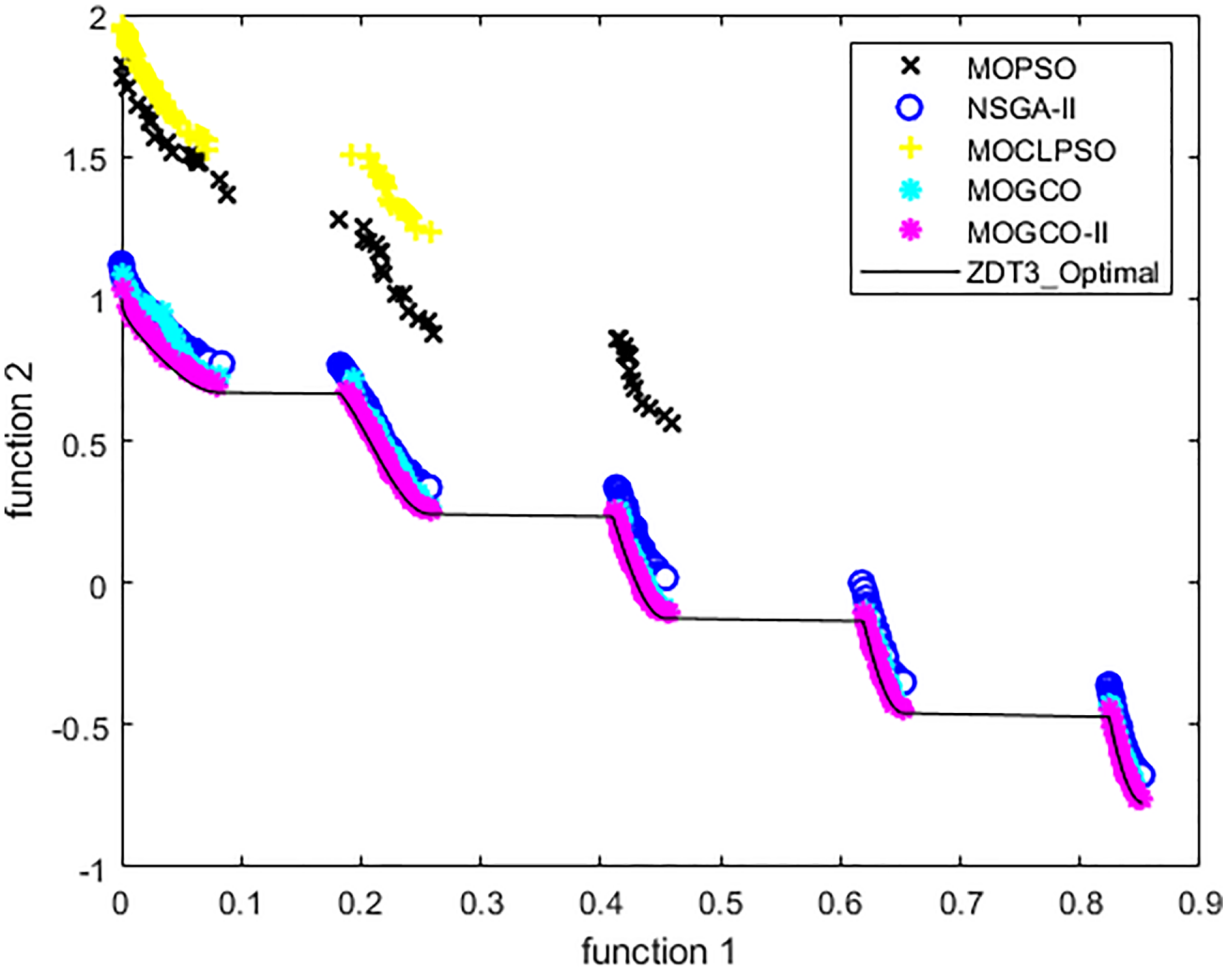
Pareto front of MOPSO, NSGA-II, MOGCO, MOCLPSO, and MOGCO-II for ZDT3.

Figure 10 presents the results produced by MOCLPSO, MOGCO, MOPSO, NSGA-II, and the proposed algorithm. The results show the best Pareto fronts have by the algorithms after 50 runs at 10,000 fitness evaluations for the test function ZDT3. In addition, the results show that MOGCO-II provides the best Pareto front, the best spread of solutions, and convergence near the actual Pareto optimal front compared to other algorithms.

### Test function 4 (ZDT4)

The ZDT4 benchmark function is used for the fourth experiment. Tables 9 and 10 show the performance metric’s comparison of MOCLPSO, MOPSO, MOGCO, NSGA-II, MOGCO, and the proposed algorithm. The tables show that MOGCO-II performs better than the existing algorithms.

**Table 9:** Results of generation distance metric for ZDT4.

FE	Algorithms	Best	Worst	Average	Median	Standard deviation
30,000	MOPSO NSGA-II MOGCO MOCLPSO MOGCO-II	1.00 × 10^–01^ 3.99 × 10^+00^ 3.84 × 10^–02^ 8.57 × 10^–02^ 1.62 × 10^–02^	3.24 × 10^–01^ 1.70 × 10^+01^ 2.09 × 10^–01^ 1.88 × 10^–01^ 2.88 × 10^–01^	1.48 × 10^–01^ 1.12 × 10^+01^ 8.17 × 10^–01^ 1.63 × 10^–01^ 8.77 × 10^–02^	1.45 × 10^–01^ 1.04 × 10^+01^ 6.92 × 10^–02^ 1.69 × 10^–01^ 5.50 × 10^–02^	3.13 × 10^–02^ 4.13 × 10^+00^ 3.84 × 10^–02^ 2.39 × 10^–02^ 7.29 × 10^–02^

**Table 10:** Results of diversity metric for ZDT4.

FE	Algorithms	Best	Worst	Average	Median	Standard deviation
30,000	MOPSO NSGA-II MOGCO MOCLPSO MOGCO-II	8.59 × 10^–01^ 8.57 × 10^–01^ 1.20 × 10^–01^ 9.79 × 10^–01^ 7.81 × 10^–02^	9.91 × 10^–01^ 1.07 × 10^+00^ 6.26 × 10^–01^ 1.01 × 10^+00^ 4.91 × 10^–01^	9.28 × 10^–01^ 9.15 × 10^–01^ 3.18 × 10^–01^ 9.97 × 10^–01^ 2.34 × 10^–01^	9.27 × 10^–01^ 9.14 × 10^–01^ 3.14 × 10^–01^ 9.98 × 10^–01^ 2.30 × 10^–01^	2.51 × 10^–02^ 3.28 × 10^–02^ 1.09 × 10^–01^ 5.47 × 10^–03^ 1.01 × 10^–01^

**Figure 11:**
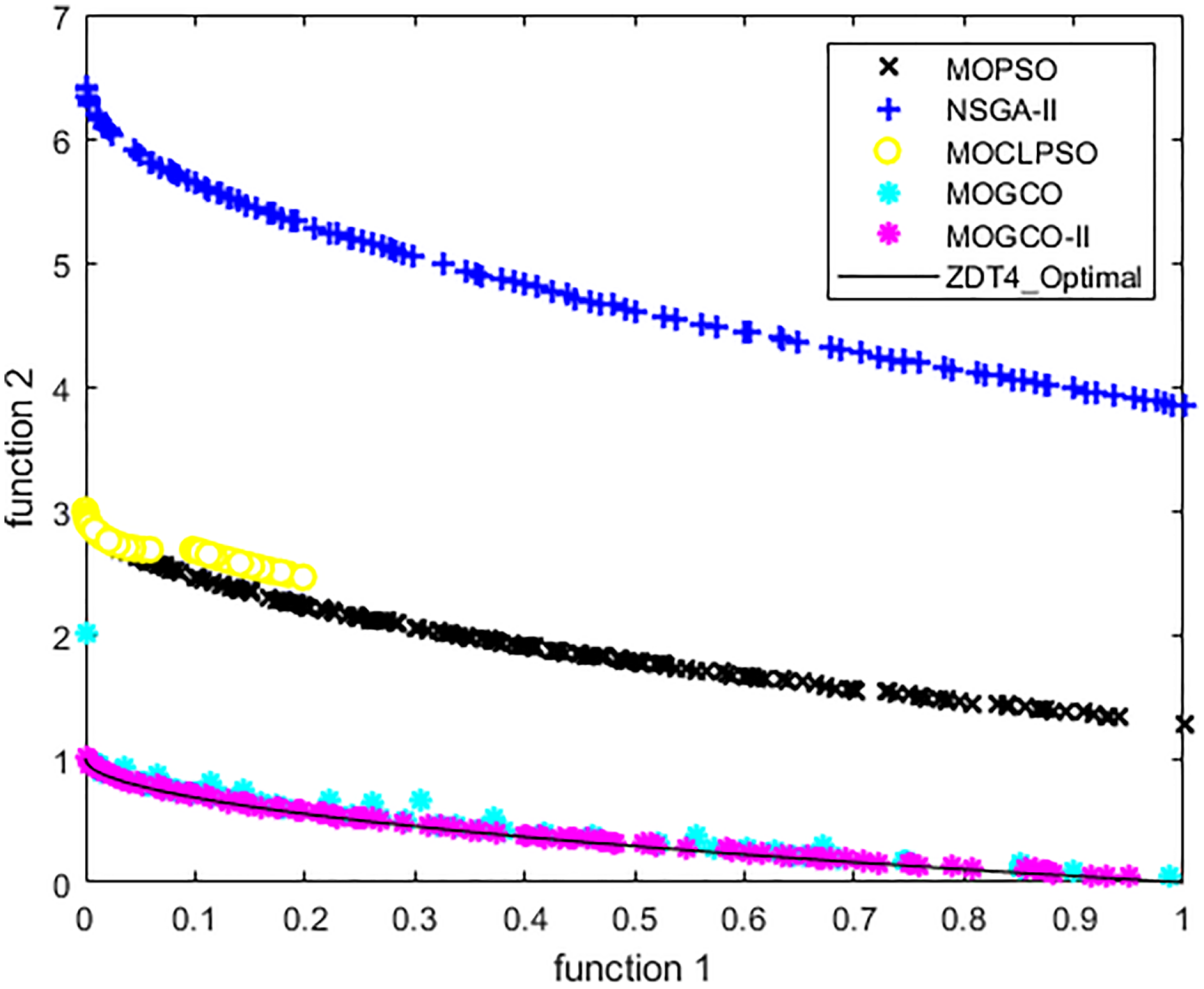
Pareto front of MOPSO, NSGA-II, MOCLPSO, MOGCO and MOGCO-II for ZDT4.

Figure 11 presents the results produced by MOCLPSO, MOGCO, MOPSO, NSGA-II, and the proposed algorithm. The results show the best Pareto fronts have by the algorithms after 50 runs at 30,000 fitness evaluations for the test function ZDT4. In addition, the results show that MOGCO-II provides the best Pareto front, the best spread of solutions, and convergence near the actual Pareto optimal front compared to other algorithms.

### Test function 5 (ZDT6)

The ZDT6 benchmark function is used for the fifth experiment. Tables 11 and 12 show the performance metric’s comparison of MOCLPSO, MOPSO, MOGCO, NSGA-II, MOGCO, and the proposed algorithm. Again, the tables show that MOGCO-II performs better than the existing algorithms. Tables 10 and 11 offer the performance metric comparison for ZDT6 of MOCLPSO, MOPSO, NSGA-II, and propose algorithm after 50 runs at 10,000 fitness evaluations for the ZDT6 test function. Again, the tables show that MOGCO-II performs better than the existing algorithms.

**Table 11:** Results of generation distance metric for ZDT6.

FE	Algorithms	Best	Worst	Average	Median	Standard deviation
10,000	MOPSO NSGA-II MOGCO MOCLPSO MOGCO-II	3.99 × 10^–01^ 3.57 × 10^–01^ 2.10 × 10^–03^ 3.56 × 10^–02^ 2.26 × 10^–03^	5.79 × 10^+00^ 5.87 × 10^+00^ 2.31 × 10^–01^ 5.21 × 10^+00^ 1.06 × 10^–01^	1.60 × 10^+00^ 1.83 × 10^+00^ 7.39 × 10^–02^ 2.96 × 10^+00^ 2.25 × 10^–02^	8.46 × 10^–01^ 1.63 × 10^+00^ 6.28 × 10^–02^ 3.68 × 10^+00^ 1.06 × 10^–02^	1.58 × 10^+00^ 1.04 × 10^+00^ 6.38 × 10^–02^ 1.93 × 10^+00^ 2.43 × 10^–02^

**Table 12:** Results of diversity metric for ZDT6.

FE	Algorithms	Best	Worst	Average	Median	Standard deviation
10,000	MOPSO NSGA-II MOGCO MOCLPSO MOGCO-II	8.72 × 10^–01^ 9.32 × 10^–01^ 2.34 × 10^–01^ 7.51 × 10^–01^ 2.17 × 10^–01^	1.37 × 10^+00^ 1.24 × 10^+00^ 1.83 × 10^+00^ 1.31 × 10^+00^ 2.70 × 10^+00^	1.16 × 10^+00^ 1.06 × 10^+00^ 8.30 × 10^–01^ 9.75 × 10^–01^ 6.71 × 10^–01^	1.18 × 10^+00^ 1.04 × 10^+00^ 8.67 × 10^–01^ 9.33 × 10^–01^ 2.91 × 10^–01^	1.35 × 10^–01^ 7.62 × 10^–02^ 4.44 × 10^–01^ 1.57 × 10^–01^ 5.63 × 10^–01^

**Figure 12:**
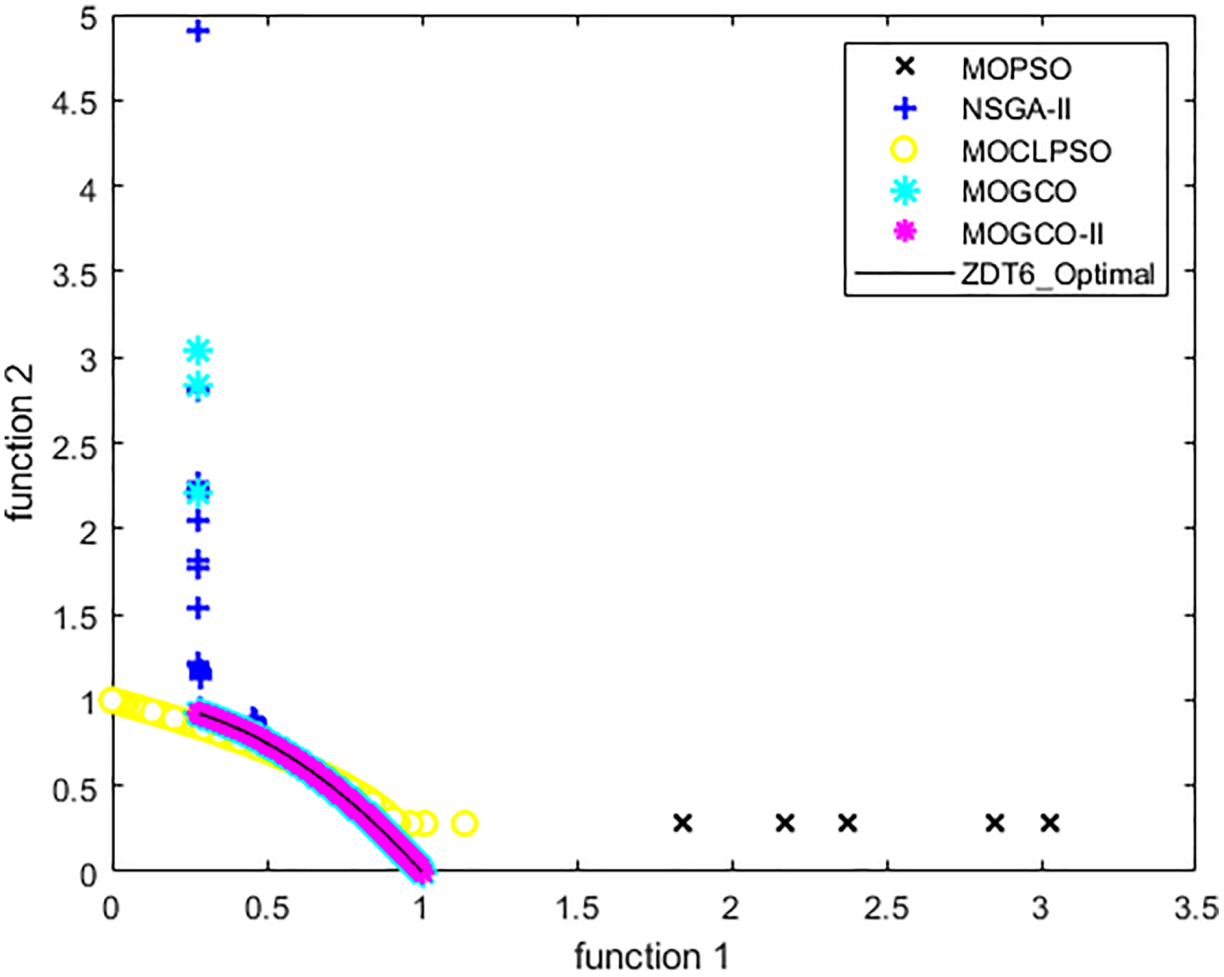
Pareto front of ZDT6 produced by MOPSO, NSGA-II, MOCLPSO, and MOGCO-II.

Figure 12 presents the results produced by MOCLPSO, MOPSO, NSGA-II, MOGCO, and the proposed MOGCO-II algorithm. The results show the best Pareto fronts of ZDT6 delivered by the algorithms after 50 runs at 10,000 fitness evaluations. This figure shows that MOGCO-II performs better.

## Optimization of Freshwater Consumption in Textile Dyeing Industry

In this work, we proposed MOGCO-II based optimization model to reduce freshwater consumption in the textile dyeing industry. For this purpose, we used the data set of Sitara Industries Faisalabad, Pakistan. We used three machines in the proposed model: Open, Pressure, and Jet. The open vessel is used for cotton fabric. The pressure vessel is used for PolyCotton fabric, and the Jet is used for both cotton and PolyCotton fabrics.

Moreover, the main parameter is the delivery date because every order must be dyed before delivery. The proposed model calculates each dyeing slot’s time, checking that any space is accessible after completing the areas for dyeing all the orders. If the slots are free, the model can adjust the new order between the free slots of pre-orders based on the fabric’s quality and color. When a new order is entered, the model reschedules all orders and regenerates the new schedule for dyeing the orders. This process is repeated whenever a new order is entered into the model. The main target of proposed model is to minimize dyeing vessels’ washing before dyeing the orders, which reduces freshwater consumption. For washing a vessel, 350 L of freshwater is required. In addition, the vessel must be cleaned twice for black color, and for all other colors, the vessel washes only once. So, if the model can minimize the washing of dyeing vessels, it can reduce freshwater consumption.

### Collection and processing of dataset

The data set is collected from Sitara Textile Industries Sargodha road Faisalabad, Pakistan. The Sitara Industry works on the exhaust method of dyeing (Jiang et al., 2010), and there are two types of cloth (Cotton and PolyCotton). The Open and Jet vessels are used for dyeing cotton and dyeing poly cotton. The Jet and Pressure vessels are used that’s why we collected the data of cotton and poly cotton for the vessels: Open, Jet, and Pressure. The parameters of the collected data are the following: order number, order place date, order delivery date, company name, cloth type, cloth quality, cloth color, color depth, dyeing time, freshwater used for washing the vessel, hard water used for dye the cloth and freshwater used for cleaning the dyed fabric.

### Results

The results prove that reducing freshwater consumption in the textile dyeing industry has remarkable benefits for the environment and could reduce wastewater treatment costs. The wastewater is treated as light polluted wastewater and high polluted wastewater. Table 13 shows the manual scheduling of the dataset collected from the Sitara Industries Faisalabad, Pakistan. In this manual scheduling, freshwater consumption is 51,100 L, as shown in Table 13. Tables 14 and 15 offer the proposed model’s optimization scheduling without and with urgent orders. In the optimization schedule, freshwater consumption is 43,050 and 44,900, reduced to 16% and 15% respectively compared to manual scheduling, as shown in Table 16.

**Table 13:** Detail of manual scheduling of orders without unexpected order.

Order #	Company name	Cloth length	Color	Color depth	Vessel #	Delivery date
1	CGU	2,000	Gray	Light	1	12/30/2019
2	CGU	2,000	Choclate	Medium	1	12/30/2019
3	CGU	2,000	NavyBlue	Medium	1	12/30/2019
4	CGU	2,000	Burgundy	Dark	1	12/30/2019
5	Camric	1,700	Brown	Medium	1	12/30/2019
6	Camric	1,700	Turqius	Medium	1	12/30/2019
7	Camric	1,700	Gray	Light	1	12/30/2019
8	Camric	1,700	Black	Dark	3	12/30/2019
9	GSItly	1,500	Beige	Light	2	12/20/2019
10	GSItly	1,500	Choclate	Medium	2	12/20/2019
11	GSItly	1,500	Green	Medium	2	12/20/2019
12	GSItly	1,500	Brown	Light	2	12/20/2019
13	GSItly	2,000	Black	Dark	3	12/20/2019
14	Eadeco	4,000	Green	Dark	1	1/30/2020
15	Eadeco	2,100	Green	Medium	1	1/30/2020
16	Eadeco	3,000	Blue	Dark	1	1/30/2020
17	Eadeco	4,000	NavyBlue	Medium	1	1/30/2020
18	Eadeco	2,500	Red	Medium	1	1/30/2020
19	Eadeco	2,500	Red	Dark	1	1/30/2020
20	Eadeco	2,000	Black	Dark	3	1/30/2020
21	LBC	2,000	Red	Light	2	1/30/2020
22	LBC	2,000	Blue	Light	2	1/30/2020
23	LBC	2,000	Yellow	Light	2	1/30/2020
24	LBC	2,500	Orange	Medium	2	1/30/2020
25	LBC	2,500	Green	Medium	2	1/30/2020
26	LBC	2,500	Gray	Medium	2	1/30/2020
27	LBC	3,000	Black	Dark	3	1/30/2020
28	STM	3,700	Brown	Light	1	1/30/2020
29	STM	3,700	Gray	Medium	1	1/30/2020
30	STM	3,700	RoyalBlue	Medium	1	1/30/2020
31	STM	3,700	NavyBlue	Medium	1	1/30/2020
32	STM	4,200	Beige	Medium	1	1/30/2020
33	STM	4,200	Gray	Medium	1	1/30/2020
34	STM	4,200	Green	Medium	1	1/30/2020
35	STM	4,200	Black	Dark	3	1/30/2020

**Table 14:** Detail of optimization scheduling of orders without unexpected order.

Order #	Company name	Cloth length	Color	Color depth	Vessel #
1	CGU	2,000	Gray	Light	1
26	LBC	2,500	Gray	Medium	2
7	Camric	1,700	Gray	Light	1
33	STM	4,200	Gray	Medium	1
29	STM	3,700	Gray	Medium	1
2	CGU	2,000	Chocolate	Medium	1
10	GSItly	1,500	Chocolate	Medium	2
3	CGU	2,000	NavyBlue	Medium	1
17	Eadeco	4,000	NavyBlue	Medium	1
31	STM	3,700	NavyBlue	Medium	1
4	CGU	2,000	Burgundy	Dark	1
5	Camric	1,700	Brown	Medium	1
12	GSItly	1,500	Brown	Light	2
28	STM	3,700	Brown	Light	1
6	Camric	1,700	Turquoius	Medium	1
8	Camric	1,700	Black	Dark	3
9	GSItly	1,500	Beige	Light	2
32	STM	4,200	Beige	Medium	1
11	GSItly	1,500	Green	Medium	2
14	Eadeco	4,000	Green	Dark	1
25	LBC	2,500	Green	Medium	2
15	Eadeco	2,100	Green	Medium	1
34	STM	4,200	Green	Medium	1
13	GSItly	2,000	Black	Dark	3
16	Eadeco	3,000	Blue	Dark	1
22	LBC	2,000	Blue	Light	2
18	Eadeco	2,500	Red	Medium	1
21	LBC	2,000	Red	Light	2
19	Eadeco	2,500	Red	Dark	1
20	Eadeco	2,000	Black	Dark	3
23	LBC	2,000	Yellow	Light	2
24	LBC	2,500	Orange	Medium	2
27	LBC	3,000	Black	Dark	3
30	STM	3,700	RoyalBlue	Medium	1
35	STM	4,200	Black	Dark	3

**Table 15:** Detail of optimization scheduling of orders with unexpected order.

Order #	Company name	Cloth length	Color	Color depth	Vessel #
1	CGU	2,000	Gray	Light	1
16	Eadeco	3,000	Blue	Dark	1
5	Camric	1,700	Brown	Medium	1
26	LBC	2,500	Gray	Medium	2
7	Camric	1,700	Gray	Light	1
33	STM	4,200	Gray	Medium	1
29	STM	3,700	Gray	Medium	1
2	CGU	2,000	Chocolate	Medium	1
10	GSItly	1,500	Chocolate	Medium	2
3	CGU	2,000	NavyBlue	Medium	1
17	Eadeco	4,000	NavyBlue	Medium	1
31	STM	3,700	NavyBlue	Medium	1
4	CGU	2,000	Burgundy	Dark	1
12	GSItly	1,500	Brown	Light	2
28	STM	3,700	Brown	Light	1
6	Camric	1,700	Turquois	Medium	1
8	Camric	1,700	Black	Dark	3
9	GSItly	1,500	Beige	Light	2
32	STM	4,200	Beige	Medium	1
11	GSItly	1,500	Green	Medium	2
14	Eadeco	4,000	Green	Dark	1
25	LBC	2,500	Green	Medium	2
15	Eadeco	2,100	Green	Medium	1
34	STM	4,200	Green	Medium	1
13	GSItly	2,000	Black	Dark	3
22	LBC	2,000	Blue	Light	2
18	Eadeco	2,500	Red	Medium	1
21	LBC	2,000	Red	Light	2
19	Eadeco	2,500	Red	Dark	1
20	Eadeco	2,000	Black	Dark	3
23	LBC	2,000	Yellow	Light	2
24	LBC	2,500	Orange	Medium	2
27	LBC	3,000	Black	Dark	3
30	STM	3,700	RoyalBlue	Medium	1
35	STM	4,200	Black	Dark	3

**Table 16:** Comparison of freshwater consumption of different scheduling techniques.

Scheduling method	The volume of wastewater (Liters)	The volume of heavy polluted wastewater (Liters)	The volume of light polluted wastewater (Liters)
Manual Scheduling	51,100	35,770	15,330
Optimization Scheduling without urgent order	43,000	30,100	12,900
Optimization Scheduling with an urgent order	44,900	31,900	13,470

Zhou et al. (2017) used a dataset of 6 months, and almost all other researchers also used a dataset of at least 6 months. In these research studies, the variation is up to 21.3%. But the proposed model used data set of just 2 months and provides a 16% improvement. If the model is applied to the 6-month dataset, the progress will be almost 32%.

## Conclusion

In this research, we proposed an algorithm that can be used for multi-objective optimization problems, particularly for optimizing freshwater consumption in the textile industry. We compared the proposed algorithm with MOPSO, NSGA-II, MOCLPSO, and MOGCO for the test functions ZDT test suite, performance metrics, and computational time. The results show that the proposed algorithm outperforms as compared to the other algorithms. This research also suggested a MOGCO-II optimization model reduce freshwater consumption in the textile dyeing industry. The variation between manual scheduling and optimization schedule is up to 35%. The limitations of the MOGCO-II algorithm are that it is only used for multiobjective optimization problems. The proposed model for optimization scheduling only deals with three vessels: open, pressure, and Jet. Furthermore, the proposed model was only tested on the data set of the exhaust textile dyeing process. Therefore, we can work on a continuous method and can be used more than three vessels in the future.

## Supplemental Information

10.7717/peerj-cs.932/supp-1 Supplemental Information 1 Code and Data Set. The data was collected manually from the Sitara Textile mills to conduct the thesis.

## Additional Information and Declarations

### Data Availability

The following information was supplied regarding data availability: The code and data set used in this project is available in the Supplemental File.
